# Efficient and cost-effective genetic analysis of products of conception and fetal tissues using a QF-PCR/array CGH strategy; five years of data

**DOI:** 10.1186/s13039-017-0313-9

**Published:** 2017-04-05

**Authors:** Celia Donaghue, Nada Davies, Joo Wook Ahn, Helen Thomas, Caroline Mackie Ogilvie, Kathy Mann

**Affiliations:** 1 Genetics Department, Viapath Analytics, Guy’s Hospital, London, SE1 9RT UK; 2 Genetics Department, Guys and St Thomas NHS Foundation Trust, London, SE1 9RT UK

**Keywords:** QF-PCR, aCGH, Products of conception, Fetal tissue, Aneuploidy, Miscarriage

## Abstract

**Background:**

Traditional testing of miscarriage products involved culture of tissue followed by G-banded chromosome analysis; this approach has a high failure rate, is labour intensive and has a resolution of around 10 Mb. G-banded chromosome analysis has been replaced by molecular techniques in some laboratories; we previously introduced a QF-PCR/MLPA testing strategy in 2007. To improve diagnostic yield and efficiency we have now updated our testing strategy to a more comprehensive QF-PCR assay followed by array CGH. Here we describe the results from the last 5 years of service.

**Methods:**

Fetal tissue samples and products of conception were tested using QF-PCR which will detect aneuploidy for chromosomes 13, 14, 15, 16, 18, 21, 22, X and Y. Samples that were normal were then tested by aCGH and all imbalance >1Mb and fully penetrant clinically significant imbalance <1Mb was reported.

**Results:**

QF-PCR analysis identified aneuploidy/triploidy in 25.6% of samples. aCGH analysis detected imbalance in a further 9.6% of samples; this included 1.8% with submicroscopic imbalance and 0.5% of uncertain clinical significance. This approach has a failure rate of 1.4%, compared to 30% for G-banded chromosome analysis.

**Conclusions:**

This efficient QF-PCR/aCGH strategy has a lower failure rate and higher diagnostic yield than karyotype or MLPA strategies; both findings are welcome developments for couples with recurrent miscarriage.

## Background

Around 15% of clinically recognized pregnancies end in miscarriage, usually toward the end of the first trimester [1], and approximately 1% of couples suffer from recurrent miscarriage (defined as three or more consecutive miscarriages). Identifying the cause of pregnancy loss is important for couples and may be critical for the management of their future pregnancies.

Traditional cytogenetic testing of miscarriage products involved culture of chorionic villi or fetal tissue, followed by G-banded chromosome analysis. This approach is labour-intensive and has a significant failure rate, especially when the sample quality is poor; in our laboratory the karyotype failure rate for these samples was approximately 30% [2]. We replaced G-banded chromosome analysis in 2007 with a combined QF-PCR/MLPA approach (samples were tested by QF-PCR for chromosomes 13, 18 21, X and Y followed, in cases with normal results, by MLPA for subtelomeres) [2]. We have now replaced this service with an extended QF-PCR assay (testing chromosomes 13, 14, 15, 16, 18, 21, 22, X and Y) followed in cases with normal results, by array comparative genomic hybridisation (aCGH).

Array analysis is now the method of choice for the identification of chromosome abnormality in postnatal samples. In our laboratory aCGH analysis is the first line test for postnatal samples [3] and for prenatal samples requiring genome-wide copy number analysis following a normal QF-PCR result [4]; ~25,000 postnatal and ~1,000 prenatal samples have been tested. Array CGH-based diagnosis has improved diagnostic yield for both postnatal and prenatal samples. However, the clinical utility of arrays for investigating pregnancy loss is still being established; the extent to which submicroscopic imbalance contributes to pregnancy loss and/or fetal abnormalities is unknown. The application of arrays for testing of fetal tissues was first described in 2004 [5]; since then there have been a limited number of published clinical cohorts [6–11]. Interpreting and reporting copy number variants (CNV) detected by aCGH in miscarriage samples is complex, given potential implications for familial testing and future pregnancies, and best practice has yet to be determined.

In addition, the UK Royal College of Obstetricians and Gyneacologists (RCOG) issued new guidelines in 2011 [12] following a review of the evidence regarding chromosome analysis of couples who suffer recurrent miscarriages [13]. It is now recommended that chromosome analysis should be performed on products of conception (POCs) and fetal tissues rather than parental blood samples. This resulted in an increase in the number of miscarriage samples received, precipitating the need for a high throughput and efficient testing strategy.

Here we describe the results from five years of our QF-PCR/aCGH testing service, including our CNV reporting criteria. This strategy has proven to be an efficient and streamlined method of testing POCs and fetal tissue samples with a lower failure rate and higher abnormality detection rate than previous testing strategies.

## Methods

### Samples

Nearly all samples were obtained from miscarriage products with a small number obtained from medical terminations or still births. Tested tissue types included chorionic villi, cord, skin, gonad, bone, muscle, lung, liver, spleen and central nervous system. Fetal tissues were always tested in preference to placental tissues, with lowest priority given to tissues from spleen, liver and lung as DNA quality is often poor from these tissues. For some pregnancies (approximately 50%) only placental material was available. The type of tissue tested was given in the report.

Gestation varied from 8 to 40 weeks. Samples where no fetal or placental material (based on morphological analysis) could be categorically identified were reported as ‘unsuitable’ and no further analysis was carried out. DNA was prepared from all samples where fetal or placental material was identified, including samples that were delayed or were macerated and which would not previously have been considered suitable for culture and karyotyping. Cultures were not routinely established.

### Testing strategy

**Fig. 1:**
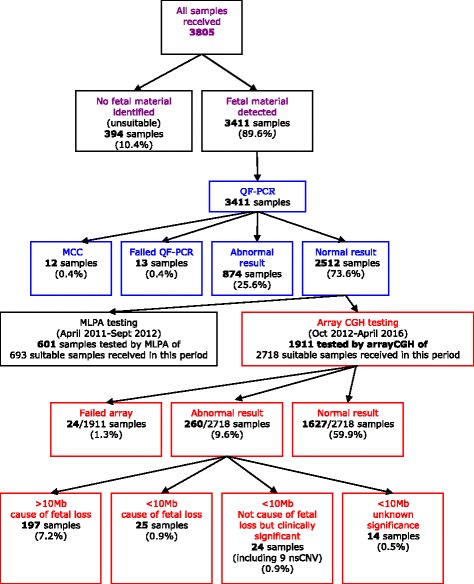
A flow chart illustrating the testing strategies and sample numbers

The testing strategy and sample numbers are summarised in Fig. 1. All samples that were not classed as unsuitable were investigated using QF-PCR assays for chromosomes 13, 16, 18, 21, X and Y following QF-PCR for aneuploidy Best Practice Guidelines [14]. Primers for chromosomes 15 and 22 were added to the multiplexes in October 2012, whilst primers for chromosome 14 were added in April 2016. QF-PCR identifies both non-mosaic and mosaic chromosome aneuploidies, molar and triploid pregnancies and maternal cell contamination (MCC). Additional cell lines, such as in cases of mosaicism and MCC, are reliably detected if they contribute at least 20% of the tested cell population. If MCC was present at a significant level (approximately 30%), such that the QF-PCR result could not be confidently interpreted, a second sample was prepared and tested. Abnormal QF-PCR results were reported and in most cases no further testing of the sample was carried out. Samples where QF-PCR detected partial chromosome imbalance were tested by MLPA or aCGH. Parental samples were requested where aneuploidies associated with a possible recurrence risk in future pregnancies were detected. Samples with a normal QF-PCR result went on to be tested using either MLPA (prior to October 2012) or aCGH (October 2012 to present). MLPA is generally considered unable to detect mosaicism whereas aCGH detects mosaicism if present at ≥20% (dependent on quality of DNA).

### DNA extraction

Forty milligrams of fetal tissue was roughly chopped or 15 mg chorionic villi was cleaned of any residual maternal decidua; these samples were incubated overnight at 56 °C with proteinase K. DNA was then extracted using a Chemagen DNA extraction robot according to the manufacturer’s instructions (Chemagen, Baesweiler, Germany). DNA was quantified using a Nanodrop spectrophotometer according to the manufacturer’s instructions (Thermo Scientific), and the DNA quality was checked by agarose gel electrophoresis. Samples with completely degraded DNA were tested by QF-PCR only and, if necessary, a second DNA sample was prepared for MLPA/aCGH analysis.

### QF-PCR testing and analysis

Amplification of microsatellite markers was carried out as described previously [15]. Details of primer sequences and multiplexes are given in Table 1. Briefly, DNA was amplified using two multiplexes that include a total of 31 markers; assay 1 contains primers for chromosomes 13, 18, 21 and 22, and assay 2, primers for chromosomes 14, 15 and 16 and the X and Y chromosomes. Supplementary markers were used as required. PCR products were separated on an ABI 3100 capillary genetic analyser, and results were analyzed using ABI Genotyper software.

**Table 1:** Marker name, location and primer sequences for assays 1 and 2

Marker Name	Location	Primer sequences 5’-3’
D13S252 F	13q12.2	**PET**-GCAGATGTACTGTTTTCCTACCAA
D13S252 R		AGATGGTATATTGTGGGACCTTGT
D13S305 F	13q13.3	**VIC**-CTAATGCAAGGAAATTTGTGG
D13S305 R		CAGCCTGTTTGAGGACCTGT
D13S634 F	13q21.33	**6FAM**-GGCAGATTCAATAGGATAAATAGA
D13S634 R		GTAACCCCTCAGGTTCTCAAGTCT
D18S976 F	18p11.31	**PET-** GAGATCCTGAACATGGAGCAG
D18S976 R		ACACTATTGGCATCCCTTGG
D18S391 F	18p11.31	**VIC**-GGACTTACCACAGGCAATGTGACT
D18S391 R		CTGGCTAATTGAGTTAGATTACAA
D18S535 F	18q12.3	**6FAM**-CAGCAAACTTCATGTGACAAAAGC
D18S535 R		CAATGGTAACCTACTATTTACGTC
D18S978 F	18q12.3	**NED**-GTAGATCTTGGGACTTGTCAGA
D18S978 R		GTCTCCCATGGTCACAATGCT
D18S386 F	18q22.1	**VIC**-TGAGTCAGGAGAATCACTTGGAAC
D18S386 R		CTCTTCCATGAAGTAGCTAAGCAG
D18S390 F	18q22.3	**NED**-GGTCAATAGTGAATATTTGGATAC
D18S390 R		CTCCAACCTCACTTGAGAGTA
D21S11 F	21q21.1	**6FAM**-TTTCTCAGTCTCCATAAATATGTG
D21S11 R		GATGTTGTATTAGTCAATGTTCTC
D21S1409 F	21q21.2	**PET**-AAGCGAAGGATTTGGATCAG
D21S1409 R		TTTGCCTCTGAATATCCCTATC
D21S1435 F	21q21.3	**6FAM**-CCCTCTCCAATTGTTTGTCTACC
D21S1435 R		ACAAAAGGAAAGCAAGAGATTTCA
D21S1411 F	21q22.11	ATAGGTAGATACATAAATATGATGA
D21S1411 R		**NED**-TATTAATGTGTGTCCTTCCAGGC
D22S873 F	22q11.21	**VIC**-GACAGAGTGACAGCCCGTCT
D22S873 R		TGGAATCTGACCTCCTCATTG
D22S528 F	22q12.2	**PET**-CTCGAGCCTGTCTCATCTCAC
D22S528 R		AGCCCAGGAGTTCTCTGTCTC
D22S685 F	22q12.3	**6FAM**-ATCTGCAAGCTCTCCAGCTC
D22S685 R		CAGTGGATCCAGGGGAAAG
D22S417 F	22q13.2	**6FAM**-AGCCTGGGAAGTTAAGACTGC
D22S417 R		ATTTTCCCATTTAGCGTTTCC
AMEL F	Xp22.2/Yp11.2	**PET**-CCCTGGGCTCTGTAAAGAATAGTG
AMEL R		ATCAGAGCTTAAACTGGGAAGCTG
TAF9L F	3p24.2/Xq21.1	AGCATCTCTGTTAAATTTAGAAATG
TAF9L R		**PET**-CAGGAAACAGCTATGACCTGCTTTTGACAGGTAGTTTTGG
DXYS267 F	Xq21.31/Yp11.2	**PET**-ATGTGGTCTTCTACTTGTGTCA
DXYS267 R		GTGTGTGGAAGTGAAGGATAG
DXYS218 F	Xp22.33/Yp11.32	**6-FAM-** AACTGAGGGGACCTGGAATG
DXYS218 R		GAATCGATTCAACCCGGGAGA
DXS981 F	Xq13.1	**6FAM**-CTCCTTGTGGCCTTCCTTAAATG
DXS981 R		TTCTCTCCACTTTTCAGAGTCA
DXS6807 F	Xp22.32	**6FAM**- TCTCCCTTATTTGTGGTTTTGC
DXS6807 R		AAAATACTCCCACCCCCAGT
DXS1283E F	Xp22.31	**NED**-AGTTTAGGAGATTATCAAGCTG
DXS1283E R		CCCATACACAAGTCCTCAAAGTGA
DXS6809 F	Xq21.33	**PET**-TTGCTTTAGGCTGATGTGAGG
DXS6809 R		CAGGTTAATTCAAGATATTTGTCA
SRY F	Yp11.31	**NED-** AGTAAAGGCAACGTCCAGGAT
SRY R		TTCCGACGAGGTCGATACTTA
DYS448 F	Yq11.223	**PET-** CAAGGATCCAAATAAAGAACAGAGA
DYS448 R		GGTTATTTCTTGATTCCCTGTG
D14S125 F	14q23.3	**6FAM**- GGTTGAATGTGGCGTGTTCCACTC
D14S125 R		CCTGGGGCTCTTAACCTCTCATCATA
D14S139 F	14q22.1	**6FAM-** TAGGCCAAAAATGCAGTCATGGGTA
D14S125 R		CTGAAAAACAAAACACAGGGGCAG
D15S1515 F	15q26.2	GAGAGATGATAAATGACAGCTACAGG
D15S1515 R		**NED**- TGGGCTATGGAAGAAACAGAG
D15S822 F	15q12	**6FAM**-CAGCAGATGTGAAGTGTGTGAA
D15S822 R		TGAGCTGCTTCTCTTTGTTGC
D16S485 F	16q22.2	**VIC**-GAAATTAAGTTTGGGATGAAACT
D16S485 R		TGAGGAACTGAGGCCATGTGA
D16S488 F	16q24.1	**VIC** -AATACAGACAGACAGACAGGT
D16S488 R		CGAAAGTGATGCCATAGACTT

Fluorescent labels added to primers are indicated: 6FAM, VIC, NED or PET (Applied Biosystems)

### MLPA testing and analysis

All MLPA procedures were carried out as described in Donaghue et al., 2010 [2].

### aCGH testing and analysis

DNA samples were labelled using CGH Labelling kit for Oligo Arrays (Enzo Life Sciences, Exeter, UK) and purified by QIA quick PCR purification kit (Qiagen, Manchester, UK) both as per the manufacturer’s instructions. Array CGH was carried out using an oligonucleotide array platform comprising 60,000 probes (Agilent, Wokingham, UK; design ID:028469), as described previously [16]. The arrays were hybridized, washed and scanned using an Agilent scanner, and output from the scanner analysed using Feature Extraction and Genomic Workbench (Agilent) in order to quantify the images and detect CNVs. Agilent ADM-2 algorithm at threshold 6 (with a 3 probe sliding window providing a median detection of 120 kb) was used to call CNVs.

A patient vs patient hybridization strategy was employed where differentially-labelled patients were hybridised against each other. For any detected imbalance, average signal intensity level for each dye was compared with intensities for that region in 10 other samples from the same array run, in order to inform “ownership” of the imbalance. Although any shared imbalance would not be detected, the risk of this was minimised by mismatching phenotypes of hybridization partners where possible and by comparing the signal intensities for each chromosome across the whole array run (typically 48 arrays) to detect any arrays where both hybridization partners carried the same whole chromosome aneuploidy. This strategy provided substantial cost savings.

All CNVs (outside established population polymorphisms) greater than 1 Mb in size were reported. For CNVs smaller than 1 Mb, only those interpreted as of fully penetrant clinical significance or as being associated with fetal abnormalities were reported.

## Results

### Sample numbers

**Fig. 2:**
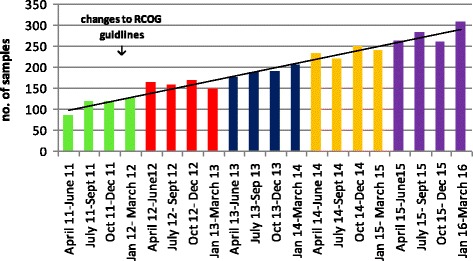
Quarterly POC and fetal tissue samples received by the laboratory

In the five year period April 2011 to March 2016, 3805 samples, including samples classed as ‘unsuitable’ were received. Figure 2 shows samples numbers received per quarter between April 2011 and March 2016. Period 1 (April 11 - March 12, *n* = 447) was prior to the implementation of the RCOG guidelines, period 2 (April 12 – March 13, *n* = 634) was the first year of the new referral criteria and was seen as a consolidation period, whilst for periods 3 (*n* = 757), 4 (*n* = 940) and 5 (*n* = 1027) all referral centres were expected to be referring samples in line with the new RCOG guidelines. This correlated with an increase in sample numbers of 130% between periods 1 and 5.

### Unsuitable samples

Three hundred ninety-four (10.4%) of samples were classed as unsuitable where no fetal or placental material was identified by morphological examination. The percentage of samples in periods 1 to 5 were 9.6%, 11.2%, 12.3, 10.2 and 8.8% respectively.

### Gestation

**Fig. 3:**
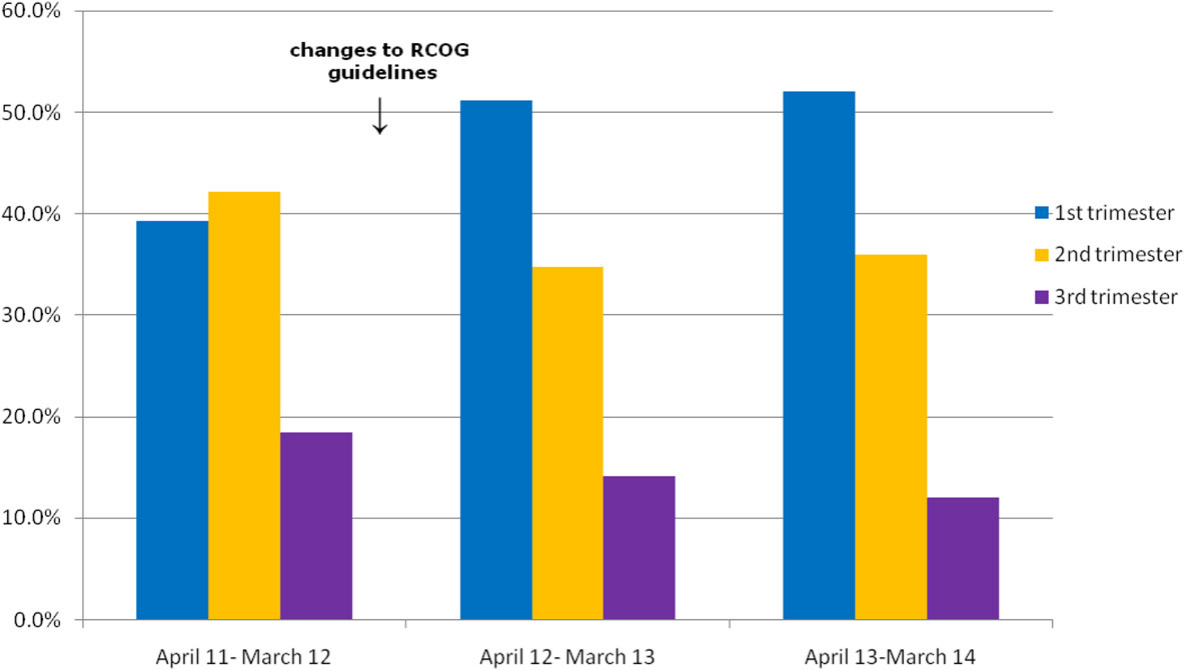
% of samples from each trimester for the first three annual periods

Gestation was available for 1126 samples, received between April 11 and March 14. For these samples the mean and median gestational age of samples over this 3 year period was 16 weeks and 14 weeks respectively. The change to the RCOG guidelines correlated with samples from the first trimester increasing from 39 to 52% between periods 1 and 3 (Fig. 3).

### Failed tests

0.4% of samples (12/3411) could not be tested due to high levels of maternal cell contamination. QF-PCR analysis failed for a further 0.4% (13/3411) of samples, whilst aCGH failed to give a result for 1.3% of samples (24/1911). The combined failure rate was 1.4% of samples (49/3411).

### Reporting times

Using the QF-PCR/aCGH testing strategy, 89% of samples were reported within 28 days; UK guidelines are 90% within 28 days [17]. This compares to 88% reported within 28 days using a QF-PCR/MLPA testing strategy [2].

### Abnormalities identified by QF-PCR

**Fig. 4:**
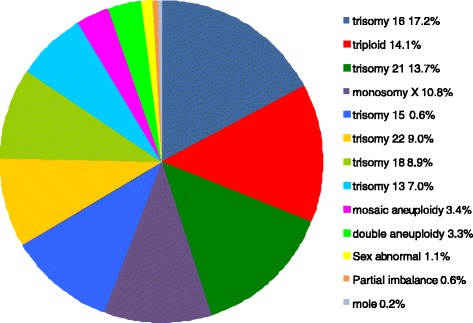
Abnormalities identified by QF-PCR

In the five year period, 874 of 3411 samples (25.6%) tested by QF-PCR were found to have a chromosome abnormality (see Fig. 4); no further testing of these samples was carried out in most cases. Testing for trisomies 15 and 22 was introduced in October 2012. Overall, trisomy 16 had the highest incidence followed by triploidy and trisomy 21. When abnormalities were detected in placental samples, the possibility of confined placental mosaicism (CPM) was considered and its significance to the miscarriage and/or fetal abnormalities was discussed.

### Abnormalities identified by aCGH

**Fig. 5:**
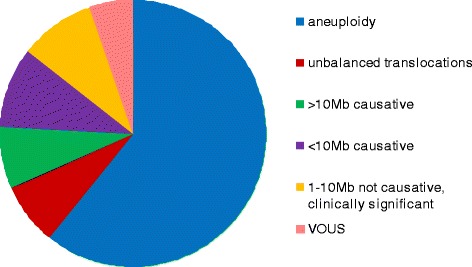
Distribution of abnormal aCGH results. VOUS (variant of unknown significance)

One thousand nine hundred-eleven samples that were normal by QF-PCR were tested by aCGH (from a total of 2718 suitable samples in this period). All chromosome imbalances >1 Mb in size were reported. In addition, imbalance <1 Mb in size associated with a fully penetrant phenotype or associated with fetal abnormalities were reported. Using these criteria, chromosome imbalance was detected and reported for 260 samples (9.6% of 2718 samples). See Fig. 5.

### aCGH abnormalities >10 Mb, causative of the miscarriage/fetal abnormalities

One hundred ninety-seven (7.2%) samples had abnormalities >10 Mb in size (Table 2); all were considered to be causative of the miscarriage and/or fetal abnormalities. Of these, 155 (5.7%) samples had whole chromosome aneuploidy including six with complex aneuploidy and nine with mosaic aneuploidy. Eighteen samples (0.7%) were found to have an unbalanced genotype indicative of a structural rearrangement; two arose from known parental rearrangements whilst sixteen samples were subsequently found to be derived from balanced parental rearrangements.

**Table 2:** Imbalance >10 Mb identified by array CGH

Imbalance	No of samples	Size (Mb)	Parental follow-up
Trisomy	136	Whole chromosome	NA
Mosaic aneuploidy	9	Whole chromosome	NA
Monosomy 21	4	Whole chromosome	4 DN
>1 chromosome aneuploidy	6		NA
Aneuploidy plus additional imbalance	4	1.445-whole chromosome	NR
Unbalanced translocations	18	1.125–120.448	all inherited from carrier parent
Tetrasomy 18p OMIM 614290	1	14.749	NR
Wolf-Hirschhorn syndrome OMIM 194190	1	39.147	NR
Pallister-Killian OMIM 601803	1	34.387	NA
6pter-p24 deletion syndrome OMIM 612582	1	11.433	NR
18p deletion syndrome OMIM 146390	1	14.749	NR
16q22 deletion syndrome OMIM 614541	1	14.560	NR
Complex imbalance	5	1.019–156.491	2 DN 3 NR
Ring/marker	3	23.215–35.232	NR
Terminal deletion	2	14.714, 23.215	NR
Interstitial deletion	1	11.218	DN
Monosomy 18p trisomy 18q	1	Whole arm	NA
2 imbalances	2	5.721–63.218	DN

*NR* not received (parental samples were requested but were not received). *NA* not applicable (parental samples were not requested as there was no indication for follow-up studies). *DN* de novo

### aCGH abnormalities <10 Mb, likely causative of the miscarriage/fetal abnormalities

Twenty-five (0.9%) samples had a reported submicroscopic abnormality (Table 3) which were either considered to be or likely to be the cause of the miscarriage and/or fetal abnormalities. These included well characterised syndrome regions eg Di-George (OMIM 188400), Williams (OMIM 194050) and Miller-Dieker syndrome (OMIM 247200) regions as well as smaller imbalances with imbalance of critical genes eg PAFAH1B1 (OMIM 601545) and HCCS (OMIM 300056) genes.

**Table 3:** Imbalances <10 Mb identified by array CGH. Imbalance considered to be the cause of pregnancy loss or fetal abnormality are shown in normal text

Imbalance	No	Size (Mb)	Parental follow-up
Williams del OMIM 194050	1	1.442	NR
Di-George del OMIM 188400	3	2.544–2.901	3 DN, 1 NR
Williams del OMIM 194050 and Di-George del OMIM 188400	1	1.416, 0.365	DN/pat
Cat Eye and VOUS OMIM 115470	1	0.524, 2.588	mat/pat
Miller-Dieker del OMIM 247200	1	6.500	mat
Mowat-Wilson del OMIM 235730	1	1.048	NR
Wolf-Hirschhorn del OMIM 194190	1	1.807	NR
RCAD del OMIM 137920	2	1.723	DN
1q21.1 dup syndrome OMIM 612475	1	1.746	NR
Saethre-Chotzen del OMIM 101400	1	5.590	NR
Translocation 5q35.3 15q26.3	1	3.083, 3.088	pat
Inv dup del 2q37.1 2q37.3	1	0.670, 8.726	NA
Complex imbalance 4p15.1 4q12 4q21.1	1	1.810, 7.528, 1.456	DN
Complex imbalance 22q12.3q13	1	0.931, 5.332 1.789	DN
ring/marker X and Y chromosomes	1	0.256	NR
20p13x1	1	4.670	NR
SRY deletion	1	4.105	DN
*1q21.1 duplication syndrome* OMIM 612475	*1*	*3.776*	*mat*
*Xq28x3,14q13.1x3*	*1*	*2.239, 1.171*	*DN*
*21q21.3q22.11x1*	*1*	*5.030*	*DN*
*17p13.3p13.2x3 Including PAFAH1B1 gene* OMIM 601545	*1*	*1.375*	*NR*
*Xp22.2x1 Including HCCS gene* OMIM 300056	*1*	*0.301*	*mat*

*Imbalance likely to be the cause of the pregnancy loss or fetal abnormality are shown in italics*. *NR* not received (parental samples were requested but were not received). *NA* not applicable (parental samples were not requested as there was no indication for follow-up studies). *DN* de novo, *VOUS* variant of unknown significance

### aCGH abnormalities <10 Mb, not causative of the miscarriage/fetal abnormalities but clinically significant

Twenty-four (0.9%) samples had a reported submicroscopic abnormality which were considered to be clinically significant but unrelated to the miscarriage and/or fetal abnormalities (Table 4).

**Table 4:** Imbalances <10 Mb identified by array CGH which are not the cause of the pregnancy loss and or fetal abnormalities but are clinically significant

Region	No.	Size (Mb)	Parental follow-up
Charcot Marie Tooth del OMIM 118200	1	1.380	NR
STS del OMIM 300747	1	1.481	NR
Sotos del OMIM 117550	1	3.594	NR
SHOX del OMIM 312865	2	0.877 1.420	NR
22q dup syndrome OMIM 608363	3	1.492–3.157	Pat, NR, NR
MSH2 OMIM 609309	1	0.103	NR
nsCNV 8p23.3, 15q13.2q13.3, 16p13.11, 16p11.2, 16p13.11	9	0.365–3.643	3 pat, 2 mat, 2 DN, 2 NR
4p16.3x3 including the ZNF141 gene OMIM 194648	1	0.469	NR
15q25.2q25.3x3	1	3.018	DN
4q27x1 including the ANXA5 gene OMIM 131230	1	0.551	Mat
Unbalanced translocation between X and Y	1	3.505, 8.460	NA
STS dup OMIM 300747	2	1.575	NR, pat
Mosaic XXX and VOUS	1	1.211	NR

*nsCNV* neurosusceptibility locus with reduced penetrance, *NR* not received (parental samples were requested but were not received). *NA* not applicable (parental samples were not requested as there was no indication for follow-up studies). *DN* de novo. *VOUS* variant of unknown significance

An additional fourteen (0.5%) samples had imbalances >1 Mb of unknown significance.

For all abnormalities with a recurrence risk, parental samples were requested for follow up studies.

## Discussion

Identifying the cause of pregnancy loss is important for couples and may have significance for the management of future pregnancies. Around 50% of early pregnancy loss is caused by sporadic chromosome aneuploidy or triploidy following meiotic or postzygotic mitotic error [18]; in these cases, the prognosis for future pregnancies is good. However, where the chromosome complement is normal, other possible reasons for miscarriage (e.g. antiphospholipid syndrome) can be considered.

We report an efficient, cost effective QF-PCR/aCGH testing strategy for POCs and fetal tissues that has a considerably lower failure rate and higher diagnostic yield compared to other approaches. The overall abnormality rate was 35.2%; 33.8% of samples were found to have a chromosome imbalance with associated fully penetrant phenotype, likely to be causative of the miscarriage or fetal abnormalities; 1.8% of these imbalances were submicroscopic (<10 Mb) and would likely not be identified by karyotype analysis. The finding of chromosome imbalance can be used to predict the risk of recurrence of both miscarriage and fetal abnormality as well as providing reasons for pregnancy loss, thus reducing further investigations.

The combined use of QF-PCR and aCGH is also more cost-effective than testing by aCGH alone. 25.6% of samples were found to be abnormal by QF-PCR; with the extended QF-PCR panel now including chromosome 14, this figure will increase. For these cases, the additional expense of the genome-wide test is avoided. In addition, QF-PCR detects triploidy (3.6% of our samples) and molar pregnancies (0.2% of our samples), neither of which are identified by MLPA or aCGH.

QF-PCR is also significantly more robust than either aCGH, MLPA or karyotype analysis; failure rates are found to be 0.4%, 1.3, 5% [2] and 30% [13] respectively. QF-PCR genotyping also identifies and quantifies MCC; an important quality check.

The oligo array platform used has an average resolution of 120 kb; however, due to the lack of data regarding the clinical significance of submicroscopic imbalance in fetal tissues and POCs, a cautious reporting approach was adopted; all imbalance >1 Mb was reported in addition to smaller regions of known fully penetrant clinical significance. In our cohort, 1.5% (40/2718) of samples were found to have a fully penetrant clinically significant submicroscopic (<10 Mb) CNV, whilst 0.3% (9/2718) were found to have a neurosusceptibility CNVs (nsCNV) >1 Mb in size. Other published cohorts found clinically significant submicroscopic imbalance in 0.6% [6], 0.8% [10], 1.6% [8] of samples, although the Levy study [10] included nsCNV and CNVs of unknown significance in this group. The patient vs patient aCGH strategy provides a significant cost reduction compared with patient vs control testing.

Use of the aCGH analysis and reporting strategy described here minimises some of the reporting and counselling complexities caused by CNVs of uncertain significance, and unexpected or incidental findings of incomplete penetrance. Relatively little is known about the genes and pathways involved in miscarriage; many CNVs identified will therefore be classed as of unknown significance and even those that are found to contain genes involved in fetal development will not be useful diagnostically or clinically without further evidence and studies [19]. If reported, CNVs of uncertain significance may require follow-up studies and raise complex counselling and ethical issues particularly regarding recurrence risks. Giving a definitive assessment of clinical significance is not possible and it is important that these results are not over-interpreted. In addition, CNVs that are linked to postnatal phenotypes may be problematic to interpret in the context of fetal abnormalities and recurrent miscarriage; for many genes more data is needed.

Neurosusceptibility CNV (nsCNV) pose further interpretation and ethical issues. As these are associated with neurodevelopmental phenotypes with incomplete penetrance they are unlikely to be the cause of the miscarriage and are therefore unexpected findings. However, as many are inherited, these findings may have clinical implications, unrelated to fetal demise, for the family and any future offspring [20]. Nondisclosure of nsCNVs is widely practised when reporting prenatal results in Europe. Our reporting policy similarly aims to minimise the disclosure of all ambiguous findings; CNVs <1 Mb are only reported if the imbalance causes a known phenotype of complete penetrance or is linked to the pregnancy loss and/or fetal abnormalities.

Imbalances associated with a well-defined and fully penetrant clinical phenotype but which are unrelated to the pregnancy loss or fetal abnormalities are further examples of unexpected findings. These are identified at low frequency; fifteen in this study. For prenatal samples there is a general consensus that if associated with a severe early onset phenotype or late onset treatable condition then these CNVs should be disclosed. A similar strategy could be applied for fetal tissue/POC samples where there is a possible recurrence risk; prenatal diagnosis may be available for future pregnancies. Given the possibility of unexpected findings, pre-test information, counselling and informed consent for aCGH testing is an essential part of the testing process; patients must be provided with information regarding both unexpected and uncertain findings and, if appropriate, nondisclosure of results.

## Conclusions

A combined QF-PCR aCGH approach is a cost-effective strategy with a higher diagnostic yield than other approaches; in more cases the cause of the miscarriage is identified and future reproductive risk determined. These outcomes are welcome developments for couples with recurrent miscarriages.
